# Electronic synergism of pyridinic- and graphitic-nitrogen on N-doped carbons for the oxygen reduction reaction†
†Electronic supplementary information (ESI) available. See DOI: 10.1039/c8sc04596h


**DOI:** 10.1039/c8sc04596h

**Published:** 2018-12-13

**Authors:** Xiaomei Ning, Yuhang Li, Jingyan Ming, Qiang Wang, Hongjuan Wang, Yonghai Cao, Feng Peng, Yanhui Yang, Hao Yu

**Affiliations:** a School of Chemistry and Chemical Engineering , Guangdong Provincial Key Lab of Green Chemical Product Technology , South China University of Technology , Guangzhou 510640 , China . Email: yuhao@scut.edu.cn ; Fax: +86 20 8711 4916 ; Tel: +86 20 8711 4916; b School of Chemistry and Chemical Engineering , Lingnan Normal University , Zhanjiang 524048 , China; c Institute of Advanced Synthesis , School of Chemistry and Molecular Engineering , Jiangsu National Synergetic Innovation Center for Advanced Materials , Nanjing Tech University , Nanjing 211816 , China; d School of Chemistry and Chemical Engineering , Guangzhou University , Guangzhou , 510006 , China

## Abstract

Nitrogen-doped carbon materials (NCs) are extensively studied for the oxygen reduction reaction (ORR). However, the nature of active sites of pyridinic nitrogen (N_P_) or graphitic nitrogen (N_G_) on NCs is still under debate. Herein, we demonstrated that the ORR activity of NCs in alkaline media depended on the electronic synergism between N_P_ and N_G_, rather than any single type of N. We measured the transferable electrons of NCs by absorption spectroscopy using 7′7′8′8-tetracyanoquinodimethane as an electron acceptor. The transferable electron amount of NCs is relevant to either N_P_ or N_G_, leading to the [N_P_] : [N_G_] ratio as an electron transfer descriptor of NCs in a reverse volcano curve manner across nineteen NCs. The similar dependence of ORR activity on the [N_P_] : [N_G_] ratio of NCs was also discovered, demonstrating the synergistic effect of N_P_ and N_G_. These results provide a new angle to understand the nature of ORR activity of NCs and optimize the ORR catalyst.

## Abstract 2

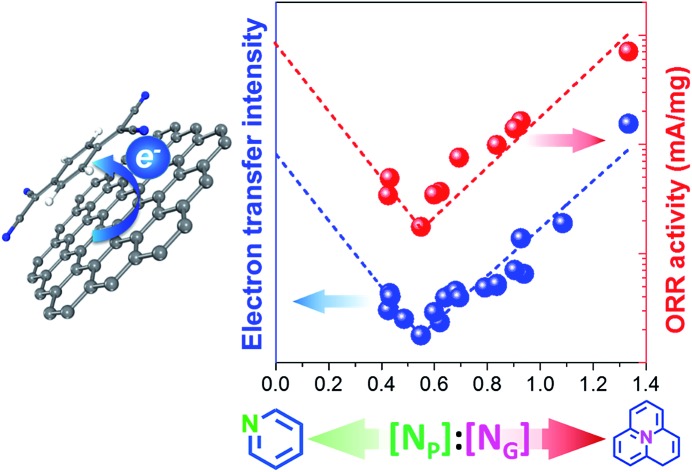
Prydinic and graphitic nitrogen both contributes to the activity for oxygen reduction reaction (ORR).

## Introduction

The oxygen reduction reaction (ORR) is essential for various cutting-edge energy conversion technologies, including metal–air batteries and fuel cells. However, the slow ORR kinetics requires a high dosage of cathodic Pt catalysts that consume two thirds of the over-potential and 90% of Pt in the whole fuel cell.^1^ Considerable efforts have been devoted to developing the low platinum group metal (PGM) or PGM-free catalysts for the ORR. Nitrogen-doped carbon materials (NCs) are promising candidates among the next generation ORR catalysts because of their low cost, high availability, and good and stable catalytic performance.^2^,^3^ N dopants modify the local electronic properties and surface chemistry of carbon materials, and therefore significantly enhance the oxygen adsorption and ORR rate.^4^,^5^ Moreover, the NCs enable the synergism with metals toward higher performance by forming M–N–C sites^6^,^7^ or metal-core@NC-shell structures.^8^–^10^

Despite the numerous contributions in this field, the active sites on NCs for the ORR are still under debate. One of the key concerns is regarding which nitrogen component, including pyridinic (N_P_), pyrrolic, and graphitic (N_G_) nitrogen substituents and nitrile and amidogen groups, contributes to the enhanced ORR catalytic activity.^11^–^13^ N_P_ and N_G_ sites have attracted extensive attention among the nitrogen sites because of their high abundance in most NCs. It has been widely documented that the N_P_ sites are responsible for the ORR activity.^5^,^14^ Yasuda *et al.*^15^ conducted the temperature-induced surface polymerization of pyridine and julolidine to produce NCs with 90% N_P_ and 80% N_G_, respectively. It was revealed that N_P_ sites are more active for the ORR than N_G_ because the former site reduces oxygen *via* a four-electron pathway whereas the latter does it *via* a two-electron process. Recently, the higher activity of N_P_ sites has been ascertained by investigating model catalysts dominated by N_P_ or N_G_ species on HOPG.^5^ Nonetheless, the N_G_ site offers considerable activity for the ORR activity as well, leading to strong correlations between the number of N_G_ sites and ORR activity in some literature studies.^12^,^16^

The controversial results imply undiscovered factors dominating the activity of NCs. It should be noted that most of the mechanistic studies employed a reductionism methodology, which attempts to correlate the activity with the amount of a single type of nitrogen to identify the so-called structure–activity relationship. To this end, model catalysts containing single N groups need to be synthesized with sophisticated routes, and it is a prerequisite to quantify their amounts *via* advanced spectroscopic technology (XPS, EELS, *etc.*) as well.^17^,^18^ However, theoretical calculations suggested that various nitrogen sites (pyridinic, pyrrolic and graphitic) are capable of adsorbing and activating oxygen, in spite of their different strengths.^19^,^20^ More importantly, the practical NCs inevitably consist of a polymorph of different nitrogen groups. They may either synergistically modulate the overall electronic structure of NCs because of the large delocalized electron distribution of the carbon matrix, or mutually affect the localized electronic properties around the N_P_ or N_G_.^21^,^22^ In fact, the mutual impact between two different dopants has recently been recognized as a significant factor to determine the catalysis of N–S/N–B/N–P co-doped carbons.^23^–^26^ Taking into account the distinctly different electronic properties of N_P_ and N_G_, it is natural to expect that the synergism between proximal N_P_ and N_G_ may lead to essentially different activity from the isolated ones, which only exist in the ideal case with extremely low site density.

Theoretical and experimental studies suggest that the carbon atom next to N_P_ or N_G_ adsorbs and activates the O_2_ in a different configuration.^5^,^11^,^27^ Zhang *et al.*^4^ have reported that the ORR activity of N-doped graphene is related to its electron spin density and atomic charge density due to the introduced N. An elaborate theoretical calculation study by Qiao and co-workers^28^ revealed that the structure of the dopant changes the density of states at the Fermi level of the carbon atoms, which modulates the capability of transferring electrons to facilitate the reduction reaction at interfaces. The differential configuration and charging of dioxygen on N_G_ and N_P_ sites have recently been proved by a DFT calculation.^29^ Similarly, the N_G_ and vacant N_P_ sites behave as electron donors and acceptors, respectively, by anchoring a platinum single metal atom, thereby the catalytic activity of the metal may be modified in redox reactions.^30^,^31^ The strong relevance of the catalysis of NCs with the electron transfer properties inspired the exploration of the dependence of electron affinity of NCs on the nitrogen dopants. Regarding N_P_ and N_G_ sites as moieties with different electron affinities, their amounts, distribution and synergism may be reflected by the overall electronic properties of NCs. By doing so, it is possible to establish a new descriptor for ORR activity of NCs, which allows for evaluation of the performance of NCs with practical compositions to understand the nature of activity of NCs.

In this work, a non-covalent conjugative interaction between NCs and 7′7′8′8-tetracyanoquinodimethane (TCNQ) was employed to investigate the electron transfer properties at the interfaces. TCNQ and its derivatives or analogues, *e.g.* tetracyanoethylene (TCNE), are commonly used as electron acceptors to study the electron donation of materials because of their small HOMO–LUMO gaps and high electron affinity.^32^,^33^ Due to the electron transfer *via* π–π interaction between TCNQ and the graphitic carbon matrix, TCNQ has been adopted as an effective molecular modifier to tune the electronic properties of graphene-based systems.^32^,^34^ More importantly, the well-developed TCNQ chemistry offers a powerful tool to quantitatively evaluate the electrons that are donated from host materials to TCNQ.

**Scheme 1:**
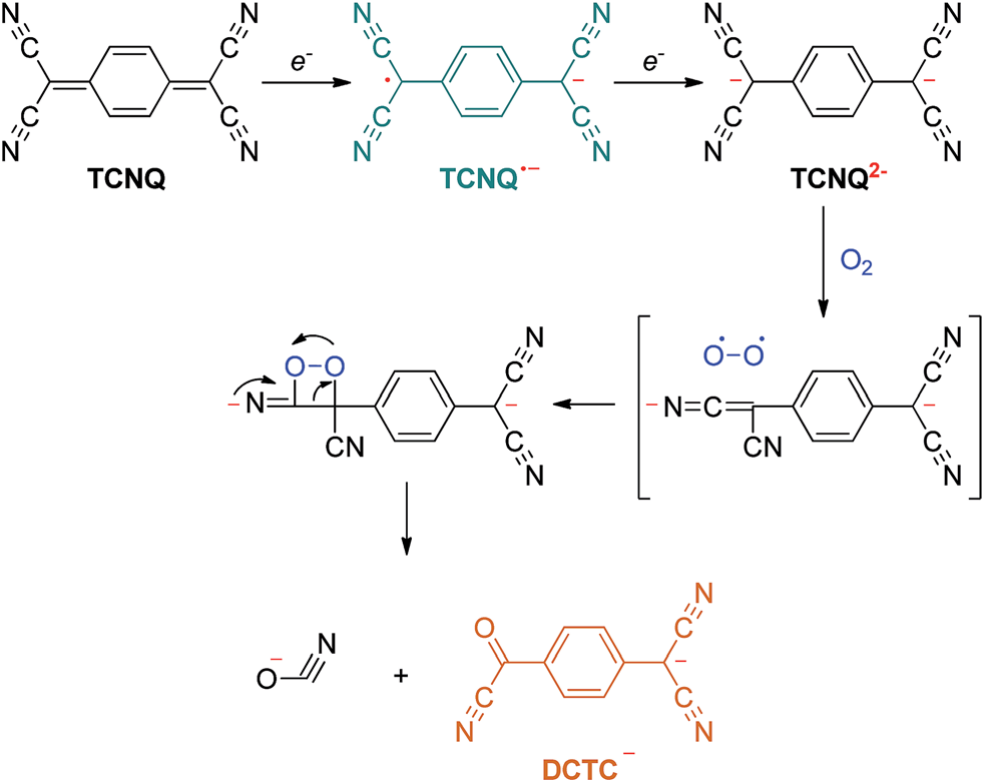
Electron transfer reaction of TCNQ.

Suchanski and Van Duyne^35^ have reported the electrochemistry of TCNQ in acetonitrile solution in the 1970s. As shown in Scheme 1, TCNQ consecutively reacts with one and two electrons to form TCNQ˙^–^ and a TCNQ^2–^ dianion. When exposed to air, a rapid oxidation reaction of the TCNQ^2–^ dianion occurs to produce an α,α-dicyano-*p*-toluoylcyanide anion (DCTC^–^), which shows a maximum at 477 nm in the absorption spectrum, leading to an orange DCTC^–^ solution. Along these lines, the reactions between NCs and TCNQ may be employed to titrate the electrons that transfer from NCs to TCNQ *via* a facile optical spectroscopic method. A similar method has been applied by Wang *et al.*^36^ to probe the transferable electrons from molybdenum oxides to supported gold nanoparticles using TCNE as the electron-accepting molecule, elucidating the electronic improvement of the MoO_*x*_ support to gold catalysis. So far, however, this approach has not been explored to quantitatively measure the electronic properties of NCs.

Herein, we demonstrate that NCs interact with TCNQ as an electron-acceptor, allowing for their transferable electrons to be counted quantitatively by absorption spectroscopy of TCNQ-derived anions. It is revealed that the transferable electron amount is highly relevant to either N_P_ or N_G_, leading to the ratio [N_P_] : [N_G_] as a new descriptor of the electron transfer, which definitely suggests a synergism between N_P_ and N_G_ on the electronic properties of NCs. For the first time, it is clearly revealed that the ORR activity of NCs in alkaline media heavily depends on the ratio [N_P_] : [N_G_] in a reverse volcano curve (V) manner, suggesting the synergistic effect of N_P_ and N_G_ on the ORR. Combined with DFT calculations, these results shed light on the dependence of ORR activity on the global electronic properties of NCs and afford a new insight into understanding the catalytic nature of NCs.

## Results and discussion

**Fig. 1:**
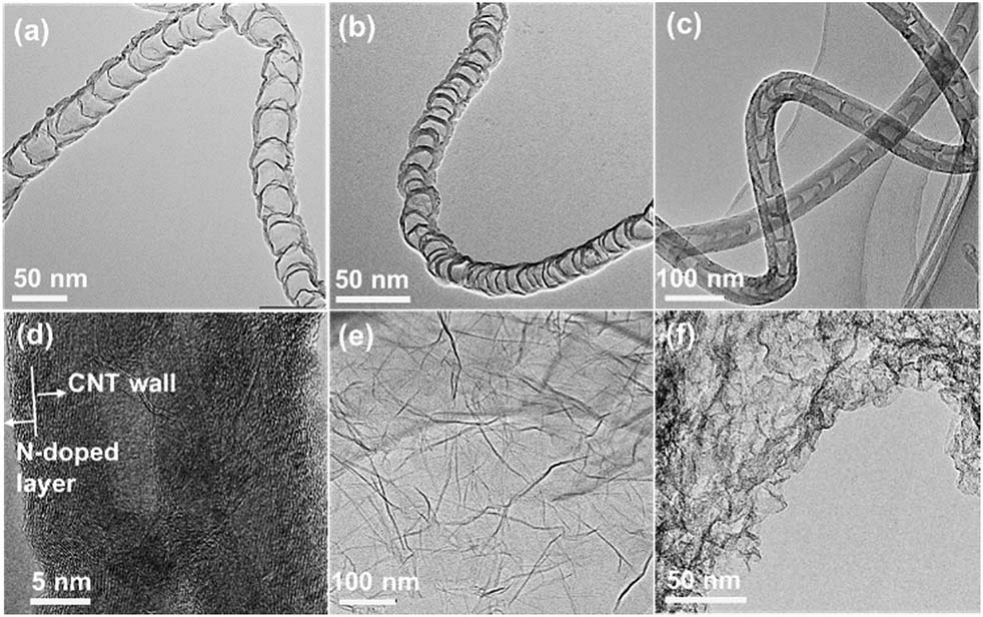
TEM images of (a) NCNTs(X–N), (b) NCNTs(A–N), (c) NCNTs(A–A), (d) N@CNTs(A-2.5), (e) N@RGO(A-2.5), and (f) NG.

A variety of NCs, with a wide spectrum of structures (graphene or carbon nanotubes) and synthesis methods, were investigated to unbiasedly reflect the effect of N dopants on electronic properties. One group of NCs investigated are N-doped carbon nanotubes (NCNTs) synthesized by a CVD method using aniline or xylene as feedstock in an argon or ammonia atmosphere.^30^,^37^ By changing the combination of feedstock and atmosphere, the amount of N dopants and thickness of CNT walls can be readily adjusted. Fig. 1(a–c) display the typical bamboo-like structure of the NCNTs.^37^ Depending on the synthesis conditions, the N content measured by XPS varied from 0.41 to 7.41 at%, as shown in Fig. S1 and Table S1.† The NCNTs prepared by the CVD method have N atoms doped in both outer and inner carbon layers.^37^,^38^ An alternative strategy was applied for the synthesis of N-doped CNTs with N dopants enriched on surfaces, denoted as N@CNTs, by a post thermolysis of pyridine on conventional CNTs.^39^Fig. 1(d) clearly shows the inner CNTs and the N-enriched surface carbon layer. By changing the duration and temperature of thermolysis, the content of N dopants can be facilely tuned (Fig. S1 and Table S1†). This approach can also be extended to other supports, *e.g.* graphene, to compare the one-dimensional and two-dimensional materials (N@RGO in Fig. 1(e)). In addition, N-doped mesoporous graphenes (NG) were also synthesized by a methane CVD method in ammonia using porous MgO as the template,^40^ and they feature a high specific surface area and N/(N + C) atomic ratio of 6.46% and 4.07% (see Fig. S1 and Table S1†), respectively.

The N_1s_ XPS spectra of the nineteen samples are displayed in Fig. S1.† Quantitative XPS analysis of nitrogen content, deconvoluted by N_P_, N_G_, pyrrolic nitrogen (N_Py_), N oxides (N_ox_), and chemisorbed nitrogen (N_ads_), as well as the ratio of [N_P_] : [N_G_] are summarized in Table S1.† For the NCNTs prepared by the CVD method, the N_G_ percentage of NCNTs(A–A)-900 synthesized in Ar is higher than that for those synthesized in an NH_3_ atmosphere, NCNTs(A–N)-900 and NCNTs(X–N)-900. Because N_G_ is more thermodynamically stable than N_P_ at higher temperatures,^41^–^43^ annealing at 1100 °C led to the decrease of the [N_P_] : [N_G_] ratio of NCNTs(A–N) from 0.94 to 0.64. For N@CNT samples, the content of surface nitrogen increased with the pyrolysis duration, from 0.41% for 1.5 h to 7.41% for 4.5 h; meanwhile, the [N_P_] : [N_G_] ratio decreased from 1.33 for 1.5 h to 0.55 for 4.5 h. Being similar to NCNTs, the [N_P_] : [N_G_] ratio of N@CNTs decreased with increasing pyrolysis temperature, accompanied by the decline of gross nitrogen content due to the decomposition and transition of N dopants. Besides, the distribution of N species can be further tuned by the atmosphere during the pyridine pyrolysis. The pyrolysis in NH_3_ considerably elevated the [N_P_] : [N_G_] ratio of N@CNTs compared to that in Ar (entries 12 and 9 of Table S1†) at the cost of decreased total nitrogen content, probably due to the etching of hydrogen from NH_3_ decomposition.^38^ The similar flexibility of the distribution of N species, represented by the [N_P_] : [N_G_] ratio, was demonstrated for graphene samples as well. Such a relatively large library of NCs covers two types of most popular nanocarbons, *i.e.* CNTs and graphene, and varies the N content from 0.41% to 7.41 at%, which may offer an opportunity to summarize the relationships between N doping and electronic properties and ORR catalytic activity.

**Fig. 2:**
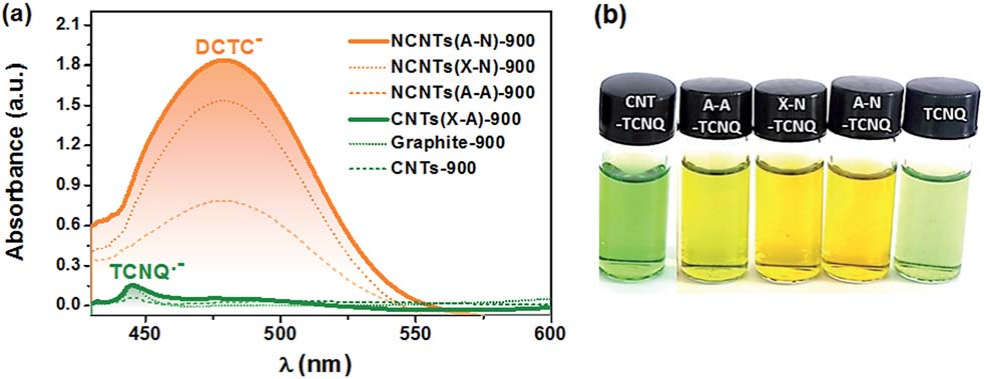
(a) Representative UV-Vis spectra of the TCNQ solutions in acetonitrile after reaction with carbon materials. The spectrum of the TCNQ acetonitrile solution that underwent the same reaction process but without carbon samples was employed as a reference. (b) Optical images of the vials of TCNQ solutions without carbon materials (rightmost) or with un-doped CNTs (leftmost) and N-doped CNTs.

The electron transfer reaction between NCs and TCNQ was employed to probe the transferable electrons *via* tracking the anions derived from the reactions in Scheme 1. The TCNQ solution in acetonitrile has a greenish color (see Fig. 2(b)) because the free radicals of TCNQ ions in acetonitrile solution absorb infrared or near-infrared light.^44^ After being thoroughly exposed to un-doped carbon materials (CNTs and graphite), the solution turned dark green, giving a weak yet distinct absorption peak at *ca.* 445 nm as shown in Fig. 2(a). This is indicative of the formation of additional TCNQ˙^–^ anions,^44^,^45^ suggesting the one-electron transfer reaction between carbon materials and TCNQ. Interestingly, the reaction between TCNQ and NCs generated completely different anionic products with a tawny color, as shown in Fig. 2(b). The optical study indicated a strong and broad absorption band centered at *ca.* 480 nm, which was absent or extremely weak in the case of un-doped carbons (Fig. 2a and S2†). The absorption can be assigned to DCTC^–^ anions, resulting from the oxidation of dianion TCNQ^2–^.^35^ Namely, the peak at *ca.* 480 nm was caused by the two-electron transfer reaction, which was only noticeable in the presence of N dopants. Hence, it was the N dopants rather than other factors (*e.g.* carbon defects) that triggered the two-electron reaction pathway between TCNQ and NCs, rationalizing the ability of NCs as an excellent material for improving electron transfer for various applications, *e.g.* optoelectronics,^46^ solar cells,^47^ and (electro-)catalysts.^48^ The different reaction mechanism may stem from the stronger adsorption of the TCNQ˙^–^ anion on NCs compared to un-doped carbons, as supported by a DFT calculation (Fig. S3†), which facilitates further electron transfer reaction of TCNQ˙^–^.

The distinct absorption peak at 480 nm enables the facile quantitation of transferable electrons on NCs through the Lambert–Beer law. Assuming that NCs are the exclusive source of electrons of DCTC^–^ anions, the absorbance at 480 nm can be a measure of transferable electrons of NCs. Taking into account the different electron transfer reaction mechanisms between N-doped and un-doped carbons, the absorbance should be normalized by the mass of NCs, since only the deposited carbon layer wrapped on surfaces contains nitrogen in the cases of N@CNTs and N@RGO, which can be determined by weighing the samples after the pyridine pyrolysis. It is defined as the intensity of electron transfer, *I*_ET_ (m g^–1^), caused by N-doping. We firstly attempted to establish the relationship between *I*_*ET*_ and the content of nitrogen measured by XPS. Unfortunately, the scattering plots of *I*_ET_*vs.* the gross nitrogen content, as well as any specific type of nitrogen (see Fig. S4†), indicate that the electron transfer reaction cannot be simply regarded as an interplay between TCNQ and isolated single N sites, and that the role of different N sites, including N_P_, N_Py_, and N_G_, may be diverse.

**Fig. 3:**
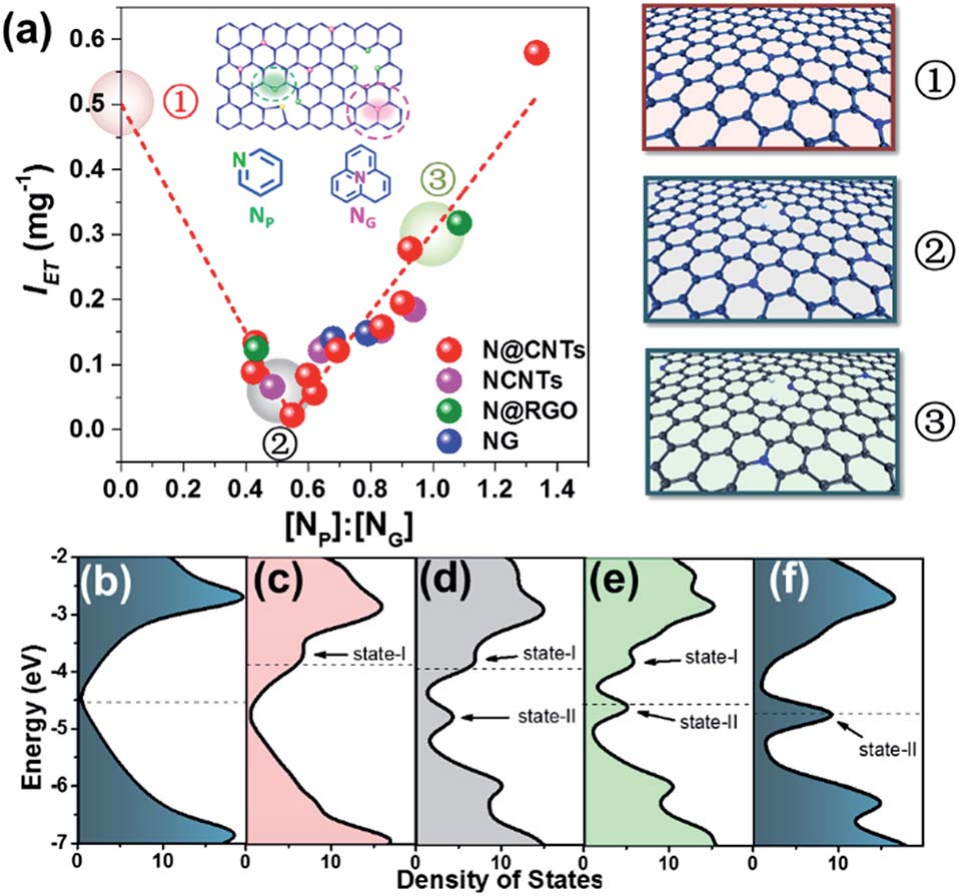
(a) Dependence of the intensity of electron transfer on the content ratio of pyridinic to graphitic nitrogen ([N_P_] : [N_G_]) measured by XPS. Three representative graphene structures, with [N_P_] : [N_G_] ratios of 0, 0.5 and 1, are illustrated in the right panel ([circle containing 1], [circle containing 2], [circle containing 3]), corresponding to the points highlighted by pink, gray and green balloons, respectively. (b), (c), (d), (e) and (f) DOS of the un-doped graphene, N-doped graphene containing only N_G_, N-doped graphene with [N_P_] : [N_G_] ratios of 0.5 and 1, and N-doped graphene containing only N_P_, respectively. The dashed lines indicate the Fermi level.

Surprisingly, a quite high relevance in a reverse volcano shape appeared when the content ratio of pyridinic to graphitic nitrogen ([N_P_] : [N_G_]) was used to correlate *I*_ET_. As shown in Fig. 3, the *I*_ET_ value decreases with the increase of the [N_P_] : [N_G_] ratio as it is smaller than 0.55. Further increasing the ratio leads to an opposite tendency. With increasing [N_P_] : [N_G_] ratio from 0.55 to 1.3, the *I*_ET_ value increases linearly from 0.02 to 0.6 mg^–1^ by 30 fold. It affords a comprehensive insight into how N dopants affect the electron transfer on surfaces of NCs. When the [N_P_] : [N_G_] ratio is small (less than 0.55 in Fig. 3(a)), there will be more transferable electrons if the sample contains more graphitic nitrogen. The opposite is also true. However, their coexistence may reduce the transferable electrons. This result rationalizes well the controversial results in the literature as interpreted in the Introduction section, demonstrating that N_P_ and N_G_ sites can both improve the electron transfer.^14^,^49^ In addition, it is impressive that the dependence is irrelevant to the structure and synthesis method of NCs across the considerably large sample library containing nineteen samples, strongly suggesting that the transferable electron may be intrinsically determined by the synergism between pyridinic and graphitic nitrogen sites. This synergism may provide a new horizon to understand the nature of active sites on NCs.

DFT calculations were employed to understand the unique allotropic synergistic effect on transferable electrons. We compared the transfer of the first electron from N-doped graphene to TCNQ, assuming that it is the rate-determining step. The calculation results suggest that the electronic interaction is stronger in the case of N-doped graphene (NG). It is revealed that the HOMO of the TCNQ-NG complex is primarily contributed by the defect carbon atom near N_P_. The valence electrons from N_G_ redistribute to the defect carbon induced by the N_P_, and then transfer to TCNQ (see Fig. S3†).

The effect of the [N_P_] : [N_G_] ratio can be illustrated by density of states (DOS) analyses through varying the numbers of N_P_ and N_G_ atoms in the graphene sheet. Three [N_P_] : [N_G_] ratios were selected to compare their electronic properties, *i.e.* 0, 0.5 and 1, as highlighted by the balloons in Fig. 3(a). Their structures are shown in the right panel of Fig. 3(a). As shown in Fig. 3(c), after introducing N_G_ atoms into graphene, a new state (marked as state-I) appears distinctly ranging from –4 to –3.5 eV compared to the pristine graphene (Fig. 3(b)). Meanwhile, the Fermi level shifts toward a more positive energy level at –3.88 eV, locating at the newly introduced state-I.^50^ In comparison, doping N_P_ atoms in graphene leads to the formation of a new energy state (marked as state-II, see Fig. 3(f)) ranging from –5 to –4.5 eV, associated with the Fermi level shifting toward a more negative energy level at –4.70 eV.^50^ Notably, despite the negative shift of the Fermi level, the electron transfer between NCs and TCNQ can also occur naturally because the calculated LUMO energy of TCNQ is –5.29 eV, meeting the requirement of orbital energy difference between NCs and TCNQ. In the presence of both N_P_ and N_G_, it can be seen that state-I is attenuated gradually as the [N_P_] : [N_G_] ratio increases, and state-II is enhanced. Importantly, the Fermi level shifts between state-I and state-II upon varying the [N_P_] : [N_G_] ratio. When the ratio is less than 0.5, the Fermi level falls into state-I, indicating that the graphitic nitrogen doping contributes primarily to the electron transfer from NCs to TCNQ. Accordingly, when the ratio is greater than 1, the Fermi level locates in state-II, showing that the pyridinic nitrogen doping dominates the electron transfer. During this evolution, the DOS at the Fermi level would firstly decrease and then increase due to the transition from N_G_-dominated to N_P_-dominated electronic properties. It should be noted that this transition passes through a DOS at the Fermi level approaching zero at a specific [N_P_] : [N_G_] ratio, where the system has the lowest electron transfer ability. Although a precise determination of this point is still unavailable because of the limitation of computational ability, the lower DOS at an [N_P_] : [N_G_] ratio of 0.5 can be indeed observed as shown in Fig. 3(d). These results are well consistent with the experimental data showing the mutual influence between N_G_ and N_P_ on the transferable electrons of NCs. It is rationalized that N_G_ and N_P_ both favor the electron transfer as isolated dopants, as widely documented in the literature.^21^,^22^ Nonetheless, their blends are not necessarily beneficial, because of the interaction between N_P_ and N_G_ with different electronic properties. In other words, the electronic properties of a single N_P_ may be different in the presence of neighboring N_G_, and *vice versa*. This circumstance may have been overlooked when investigating model catalysts previously, thus resulting in the controversial results.

Bearing this synergism between N_P_ and N_G_ in mind, the ORR performance of the NCs in alkaline media was revisited. Considering the complexity of the ORR involving multiple elementary steps, the catalytic investigation was restricted to the ten N@CNTs samples, in which the CNT inner core affords a conductive network while the surface N-doped carbon layer more efficiently catalyzes the ORR.^39^ By doing so, it is attempted to keep the structure, surface area, and porosity of electro-catalysts as constant as possible, since they have been widely documented to be potent to ORR activity.^16^,^51^,^52^ Linear sweep voltammetry (LSV) tests on an RDE at different rotating speeds were conducted for extracting the kinetic parameters of the ORR through the Koutecky–Levich (K–L) equation:*j*^–1^ = *j*_l_^–1^ + *j*_k_^–1^ = (0.62*n*FCD^2/3^*ν*^–1/6^*ω*^1/2^)^–1^ + *j*_k_^–1^.

**Fig. 4:**
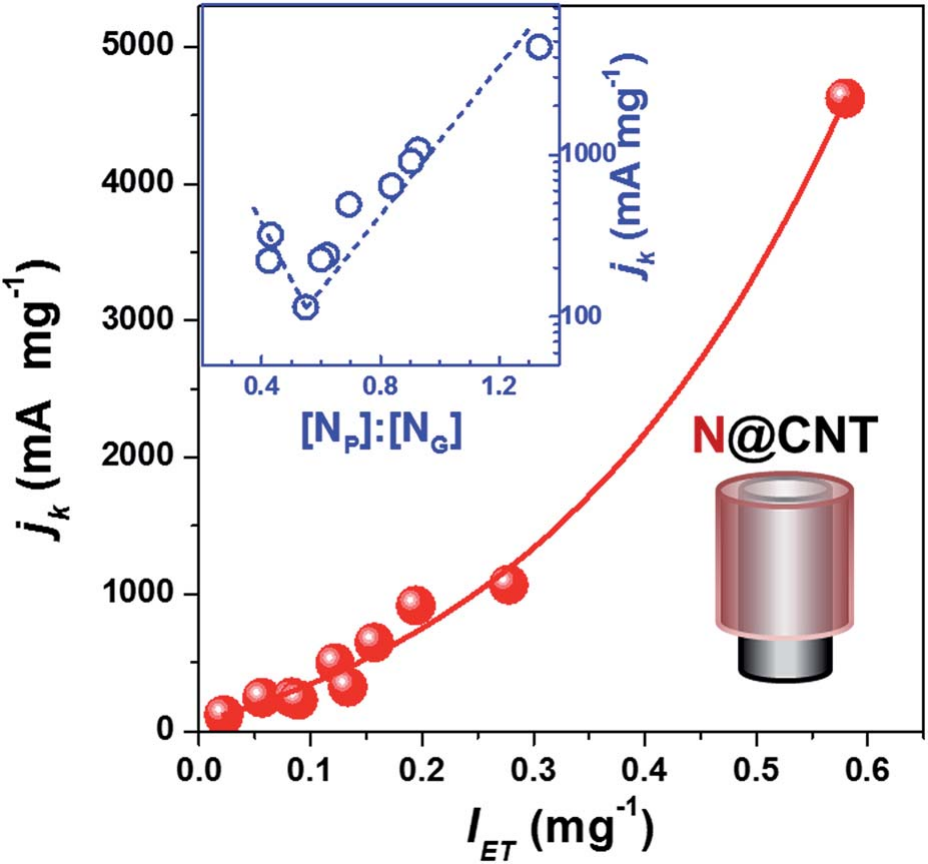
Dependences of kinetic current densities (*j*_k_) of the ORR at –0.7 V *vs.* Ag/AgCl on the intensity of electron transfer and [N_P_] : [N_G_] ratio (inset) of N@CNTs with a coaxial cable structure as shown in the lower right panel. *J*_k_ was obtained according to the Koutecky–Levich (K–L) equation, and normalized by the mass of NCs. Reaction conditions: the LSV tests were performed in O_2_-saturated 0.1 M KOH from –1 V to 0.2 V at a scan rate of 5 mV s^–1^ under different rotation rates.

The kinetic current density (*j*_k_), which has been recommended as a descriptor of ORR activity,^53^,^54^ can be obtained by fitting the experimental data in the K–L plots (*j*^–1^*vs. ω*^–1/2^) as shown in Fig. S6.† The electron transfer number of N@CNTs determined by the Koutecky–Levich (K-L) equation is within the range from 4.2 to 2.2. As a simplified prediction, the ORR activity should benefit from the stronger electron donating ability of the catalyst because oxygen accepts electrons to produce OH^–^ or H_2_O_2_. Fig. 4 displays the *j*_k_ value at –0.7 V as a function of intensity of electron transfer (*I*_ET_) defined by the absorption of TCNQ at 480 nm. Depending on the amount and type of N-dopant wrapped on CNTs, the *j*_k_ varies in a wide range from 114.6 to 4623.5 mA mg^–1^ almost by 40 fold enhancement, demonstrating the improvement of ORR activity by the N-doping strategy. More importantly, we revealed the clear relationship between *j*_k_ and *I*_ET_. The *j*_k_ at –0.7 V monotonically increases with *I*_ET_ as shown in Fig. 4, suggesting the crucial role of electron transfer on catalyst surfaces in the ORR activity. A similar dependence between *j*_k_ and *I*_ET_ was obtained using the *j*_k_ at –0.6 V and –0.5 V from the K–L plots (see Fig. S7†). In addition, the Tafel equation was applied to evaluate the ORR performance of N@CNTs (see Fig. S8† for the Tafel curves). The Tafel slope declines with increasing *I*_ET_ from 105 to 70 mV dec^–1^ (Fig. S9†). This result implies that the strong electron transfer may reduce the over-potential, as shown in Fig. S10.†

To understand the structure–activity relationship of NCs, we correlated *j*_k_ with the gross nitrogen content, as well as any specific type of nitrogen, but scattering plots were obtained again (see Fig. S11†). However, a reverse volcano-type curve can be plotted when summarizing the structure–activity relationship using the [N_P_] : [N_G_] ratio as a descriptor, as displayed in the inset of Fig. 4 and S7.† The high content of either N_G_ or N_P_ can improve the activity. *j*_k_ has a minimum at an N_P_ : N_G_ ratio of 0.55, where the N@CNTs have the lowest amount of transferable electrons as mentioned earlier. The highest ORR activity is reached over the sample with the highest [N_P_] : [N_G_] ratio, which is consistent with the widely accepted conclusion that pyridinic nitrogen is the most active site for the ORR. However, our results indicate that the graphitic nitrogen can be alternative active sites when the content of pyridinic nitrogen or the [N_P_] : [N_G_] ratio is low. Nevertheless, we emphasize that the dependence of ORR activity on the [N_P_] : [N_G_] ratio is confirmed for the NCs with a similar synthesis and structure, and other factors, such as micropores, nitrogen content, and conductivity, may influence the activity.

The above results provide a new insight into the nature of the active sites of the ORR on NCs. Differing from conventional elucidation, our results emphasize the synergistic effect of different types of N-dopants on the electron transfer and ORR activity. The activity of nitrogen-containing active sites may be influenced by other N-dopants. Because of the different electronic properties of N_P_ and N_G_, their co-existence may reduce the electron transfer and thus the ORR activity, as evidenced by the reverse volcano curve ascribed to the [N_P_] : [N_G_] ratio. This suggests that the content of N_P_ and N_G_ has to be synchronously controlled for the rational design of NCs as ORR catalysts. Moreover, the design, synthesis and characterization of the graphene-like unit containing different nitrogen configurations could be interesting, which may lead to a new paradigm for synthesizing metal-free electrocatalysts.

## Conclusions

In summary, we developed a method to measure the transferable electrons of NCs using TCNQ as an electron accepting molecule. It was found that the strong electronic interaction between NCs and TCNQ facilitated the two-electron reduction reaction of TCNQ to produce DCTC^–^ anions, which can be quantitatively analyzed by absorption spectroscopy with a characteristic peak at 480 nm, allowing for the transferable electrons to be counted as a measure of the electron donating ability of NCs. By this facile technique, it was uncovered that the intensity of electron transfer on NCs depended on the [N_P_] : [N_G_] ratio in a reverse volcano curve across a wide spectrum of NCs. When these NCs were subjected to the ORR in alkaline media, a similar relationship between the kinetic current density and the [N_P_] : [N_G_] ratio was observed. This result sheds light on the roles of N_P_ and N_G_ in determining the electronic properties and ORR activity of NCs. Namely, isolated N_P_ or N_G_ can both improve the ORR activity, while their interaction results in lower electron donation and ORR activity, as revealed by experimental and theoretical data. Our results provide a new horizon to survey and optimize the activity of metal-free NC electrocatalysts. The insight into the synergistic effect of N_P_ and N_G_ on electronic properties also paves a new avenue to enhance other cutting-edge applications based on NCs, such as supports of metal catalysts, co-catalysts for photo-electro-catalysis, dye-sensitized solar cells, *etc.*

## Experimental and computational methods

### Catalyst preparation

#### Synthesis of NCNTs and NGs

NCNTs were synthesized by a chemical vapor deposition (CVD) of aniline or xylene over an FeMo/Al_2_O_3_ catalyst in a horizontal tube furnace, as detailed in our previous reports.^30^,^55^ The residual catalyst in the as-synthesized NCNTs was removed using HF solution (v/v 15%) for 6 hours at room temperature. The washed NCNTs were filtered, dried and ground. The obtained samples are marked as NCNTs(A–N), NCNTs(A–A), and NCNTs(X–N), where A–N, A–A and X–N stand for aniline–NH_3_, aniline–Ar, and xylene–NH_3_, respectively. Besides, the un-doped CNTs were synthesized using xylene in Ar, denoted as CNTs(X-A).

The N-doped graphene (NG) was synthesized by a CVD method with CH_4_ as a carbon source and introduced NH_3_ as a nitrogen source over the MgO catalyst in a horizontal tube furnace.^40^,^56^ The NG was obtained after CH_4_ cracking at 700 °C for 20 min. The sample was further purified using HCl reflux, and then filtered, dried and ground.

#### Synthesis of N@CNTs and N@RGO

N@CNTs was synthesized *via* a surface nitrogen modification strategy by pyridine pyrolysis.^39^ In brief, CNTs (purchased from Shenzhen Nanotech Port Co., Ltd.) were put in a horizontal tube furnace at 760 °C in Ar or NH_3_ at 200 N cm^3^ min^–1^. Pyridine was injected using a syringe pump at a rate of 1.5 mL h^–1^ for 1.5 h, 2.5 h, 3.5 h, and 4.5 h, respectively. After injection, the furnace was kept at 760 °C for 10 min to ensure that the residual pyridine reacted. After cooling, washing with alcohol and acetone and drying, the obtained samples are denoted as N@CNTs(A-*t*) (*t* = 1.5, 2.5, 3.5, and 4.5 h) and N@CNTs(N-2.5), where A and N stand for Ar and NH_3_, respectively.

Graphite oxide (GO) was synthesized through a modified Hummers' method, as detailed in ref. 57 and 58. The GO was further reduced in 10% H_2_/Ar at 700 °C for 2 h. The same pyridine pyrolysis was conducted on the reduced GO (RGO) in Ar or NH_3_ for 2.5 h. The resulting samples are denoted as N@RGO(A-2.5) and N@RGO(N-2.5), respectively.

#### Purification and annealing treatment

Before reacting with TCNQ, all the carbon materials were degassed at 200 °C in 100 N cm^3^ min^–1^ Ar for 2 h to remove residual moisture and adsorbates.

NCs were further annealed at 800 °C, 900 °C, or 1100 °C in Ar gas at 100 N cm^3^ min^–1^ for 2 h, to vary the nitrogen content and distribution. The resulting samples are denoted as NCNTs-*T*, NG-*T*, or N@CNTs-*T* (*T* = 800/900/1100 °C).

### Catalyst characterization

X-ray photoelectron spectroscopy (XPS) analysis was conducted using a Kratos Axis Ultra (DLD) spectrometer with an AlKα X-ray source. The calibration peak of C 1s was defined at 284.6 eV. The morphology of the samples was observed by transmission electron microscopy (TEM) with a JEOL JEM2010 microscope operating at 200 kV. UV-Vis absorption spectroscopy (SHIMADZU, UV-2450) measurement was performed using a TCNQ acetonitrile solution as a reference.

### Computational methods

The periodic pure graphene and nitrogen-doped graphene were initially optimized with the DMol3 package.^59^ The optimization was achieved with the Perdew–Burke–Ernzerhof (PBE)^60^ exchange–correlation functional under the DNP basis set (nearly equivalent to 6-31G** in Gaussian). The dispersion correction contributed by Grimme was considered during calculations.^61^ The *k*-point was set to 8 × 8 × 1 for the self-consistent field (SCF) procedure, and 12 × 12 × 1 for the density of states calculation. The conductor-like screening model (COSMO) with a permittivity of 37.5 was invoked to mimic the acetonitrile solvent environment.^62^ The global orbital cutoff was 4.5 Å.

The primitive cell of graphene was extended to a 6 × 6 × 1 supercell. Avoiding the interaction between neighboring lattices, 25 Å was set as the vacuum layer along the direction perpendicular to the graphene surface. After optimization, two carbon atoms on average were replaced with nitrogen atoms to build the N-doped graphene, including a N_G_ atom and a N_P_ atom. Notably, the topological defect was inevitably generated once N_P_ was introduced onto the graphene surface. Herein, the defect carbon atoms were saturated with hydrogen. The calculations mentioned above were adopted for geometry optimization and electronic structure determination.

The optimized structures were further calculated with Quantum ESPRESSO 6.2 to evaluate the TCNQ adsorption energy. Herein, the GBRV ultrasoft pseudopotentials based on the PBE exchange–correlation functional were invoked. Global kinetic energy cutoffs were set to 35 Ry for the involved elements of H, C and N. Kinetic energy was set to 280 Ry for charge density and potential. Grimme's DFT-D3 was included as the long-range dispersion correction method. The electronic smearing value was 0.002 Ry (0.027 eV). The *k*-point setting was 8 × 8 × 1, the same as that for the calculations in DMol3.

### Reactions

#### Electron transfer reaction between carbons and 7′7′8′8-tetracyanoquinodimethane (TCNQ)

The samples were exposed to 7.65 mM TCNQ acetonitrile solution to trigger the electron transfer reaction. Before that, the acetonitrile was firstly dehydrated with an activated molecular sieve for 24 h. 1.5 mg mL^–1^ carbon was dispersed in the TCNQ acetonitrile solution in a vial through ultrasonic treatment. The suspension was stirred at 80 °C for 20 min to facilitate the reaction. After cooling the suspension to room temperature, it was centrifuged to obtain a liquid, which was further analyzed by UV-Vis spectroscopy.

#### Oxygen reduction reaction (ORR)

Electrochemical tests were carried out in a three-electrode cell connected to a computer-controlled workstation (CH Instruments 760D) equipped with a rotation speed controller (Pine Instrument Co., AFMSRCE). A Ag/AgCl electrode saturated with KCl and a graphitic electrode were employed as reference and counter electrodes, respectively. A glassy carbon (GC, 5.0 mm diameter) disk was used as the working electrode. The sample ink was obtained by ultrasonically dispersing the catalyst powder (1 mg) into the dispersion liquid, which consisted of Nafion (5 wt%), isopropyl alcohol and distilled water with a volume ratio of 3 : 20 : 77. 15 μL ink was cast on the surface of the GC disk for obtaining the Rotating Disk Electrode (RDE). Linear sweep voltammetry (LSV) tests were performed in 0.1 M KOH solution saturated with oxygen from –1 V to 0.2 V at a scan rate of 5 mV s^–1^ with different rotation rates (200, 400, 600, 900, 1200, and 1600 rpm).

## Conflicts of interest

There are no conflicts to declare.

## Supplementary Material

Supplementary information
