# Supplementary material for: Electronic synergism of pyridinic- and graphitic-nitrogen on N-doped carbons for the oxygen reduction reaction
Source: Chem Sci. 2018 Dec 13;10(6):1589–96. doi: 10.1039/c8sc04596h (PMC6371757; doi:10.1039/c8sc04596h)
Supplement: Supplementary file 1 [file SC-010-C8SC04596H-s001.pdf]

Electronic Supplementary Material (ESI) for Chemical Science.
This journal is © The Royal Society of Chemistry 2018

Supporting Information

Electronic Synergism of Pyridinic- and Graphitic-Nitrogen on N-doped Carbons for Oxygen Reduction Reaction

Xiaomei Ning [a,b†], Yuhang Li [a†], Jingyan Ming [a], Qiang Wang [c], Hongjuan Wang [a], Yonghai Cao [a], Feng Peng [d], Yanhui Yang [c], Hao Yu [a*]

[a] *School of Chemistry and Chemical Engineering, Guangdong Provincial Key Lab of Green Chemical Product Technology, South China University of Technology, Guangzhou 510640, China*

[b] *School of Chemistry and Chemical Engineering, Lingnan Normal University, Zhanjiang 524048, China*

[c] *Institute of Advanced Synthesis, School of Chemistry and Molecular Engineering, Jiangsu National Synergetic Innovation Center for Advanced Materials, Nanjing Tech University, Nanjing 211816, China*

[d] *School of Chemistry and Chemical Engineering, Guangzhou University, Guangzhou, 510006, China*

[*] To whom correspondence should be addressed.
yuhao@scut.edu.cn (H Yu), Tel: +86 20 8711 4916, Fax: + 86 20 8711 4916.
† They contribute equally to this work.

Table S1 Properties of the NCs investigated in this work.

Sample	S_{BET} (m_2/g)	N/(C + N) (%)	N_P (%)	N_{Py} (%)	N_G (%)	N_{ox} (%)	N_{ads} (%)	**[N_P]:[N_G]**
NCNTs(A-A)-900	-	3.14	0.78	0.07	1.61	0.38	0.30	0.48
NCNTs(X-N)-900	-	1.75	0.48	0.10	0.73	0.18	0.27	0.66
NCNTs(A-N)-900	-	2.46	0.86	0.03	1.03	0.24	0.31	0.83
NCNTs(A-N)-800	-	2.57	0.91	0.08	0.97	0.15	0.47	0.94
NCNTs(A-N)-1100	-	2.25	0.62	0.07	0.98	0.34	0.24	0.64
NG-800	705	6.46	1.73	1.32	2.55	0.43	0.43	0.68
NG-900	-	4.07	1.21	0.80	1.53	0.33	0.20	0.79
N@CNTs(A-1.5)	78	0.41	0.12	0.06	0.09	0.07	0.08	1.33
N@CNTs(A-2.5)	68	4.75	1.49	0.21	2.15	0.52	0.38	0.69
N@CNTs(A-3.5)	64	6.56	1.99	0.27	3.21	0.78	0.31	0.62
N@CNTs(A-4.5)	43	7.41	2.09	0.23	3.81	0.83	0.45	0.55
N@CNTs(N-2.5)	78	2.13	0.63	0.41	0.68	0.20	0.20	0.93
N@CNTs(A-2.5)-800	69	4.51	1.24	0.27	2.07	0.59	0.33	0.60
N@CNTs(A-2.5)-900	72	2.34	0.49	0.25	1.15	0.23	0.22	0.43
N@CNTs(A-2.5)-1100	75	0.78	0.13	0.11	0.32	0.07	0.15	0.42
N@CNTs(N-2.5)-800	80	1.04	0.26	0.27	0.29	0.12	0.09	0.90
N@CNTs(N-2.5)-1100	81	0.47	0.12	0.10	0.14	0.06	0.04	0.84
N@RGO(A-2.5)	527	5.53	1.30	0.36	3.00	0.68	0.20	0.43
N@RGO(N-2.5)	515	5.24	1.92	0.74	1.77	0.50	0.33	1.08

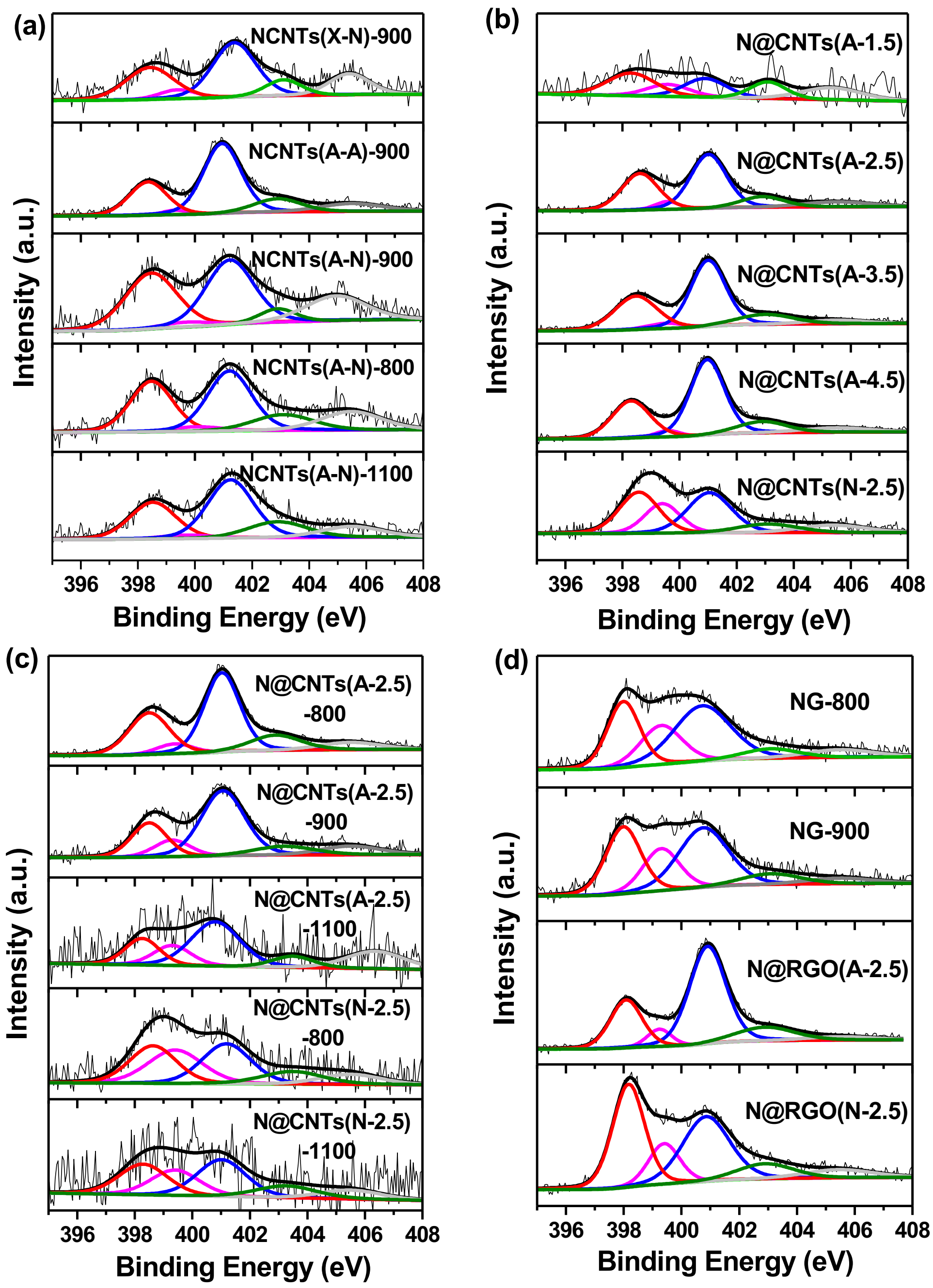

Figure S1. N1s XPS spectra of (a) NCNTs, (b) N@CNTs varying deposition time, (c) samples of N@CNTs(A-2.5) and N@CNTs(N-2.5) annealed at high temperatures, and (d) graphene-based samples, respectively.

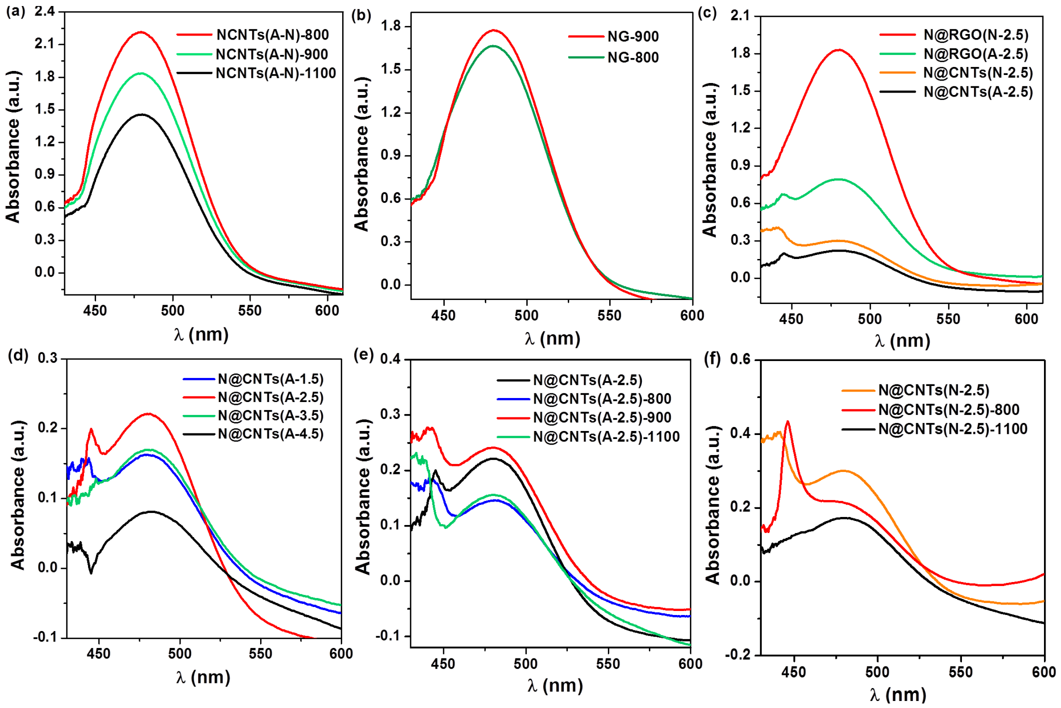

Figure S2. UV-Vis spectra of the TCNQ solutions in acetonitrile after reacted with N-doped carbon materials. In this work, we used the spectrum of TCNQ acetonitrile solution as reference. Since the TCNQ reference contains a certain amount of its radical anion ($TCNQ^{\cdot-}$), the negative peak at ca. 450 nm signifies the less amount of $TCNQ^{\cdot-}$ anions after reaction with carbon materials, probably due to the transformation of $TCNQ^{\cdot-}$ anions to $TCNQ^{2-}$ dianions through a one-electron transfer reaction.

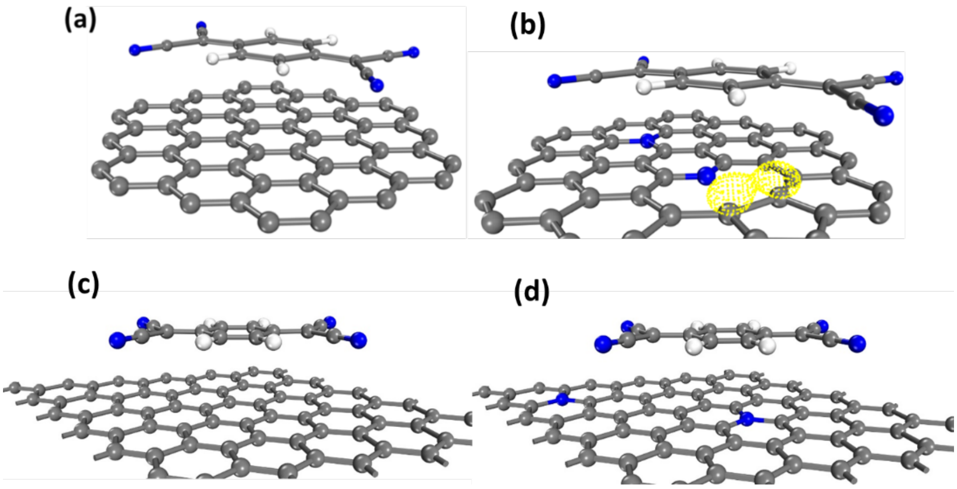

Figure S3. The optimized structures of (a) PG-TCNQ, (b) NG-TCNQ, (c) PG-TCNQ$\bullet^{-}$ and (d) NG-TCNQ$\bullet^{-}$. The TCNQ molecule is adsorbed on the pristine graphene (PG-TCNQ) and nitrogen-doped graphene (NG-TCNQ) containing equal number of N_P and N_G atoms, respectively. For PG-TCNQ in the ground state, the Fermi level is -4.470 eV and the LUMO of TCNQ is -3.253 eV. For NG-TCNQ in the ground state, the Fermi level is -3.918 eV and the LUMO of TCNQ is -3.119 eV. After the identification of wavefunctions, it was found that the HOMO of NG-TCNQ is primarily contributed by the defect carbon atoms near the N_P atom, highlighted by the yellow zone in (b).

The adsorption energy of TCNQ on graphene (a) and N-doped graphene (b) are -0.881 and -1.795 eV, respectively, indicating the stronger adsorption on N-doped graphene. Meanwhile, the adsorption energy of TCNQ$\bullet^{-}$ on graphene (c) and N-doped graphene (d) are +0.645 and -0.139 eV, respectively. This result indicates that the anion cannot be stabilized on pristine graphene, but can be weakly adsorbed on N-doped graphene.

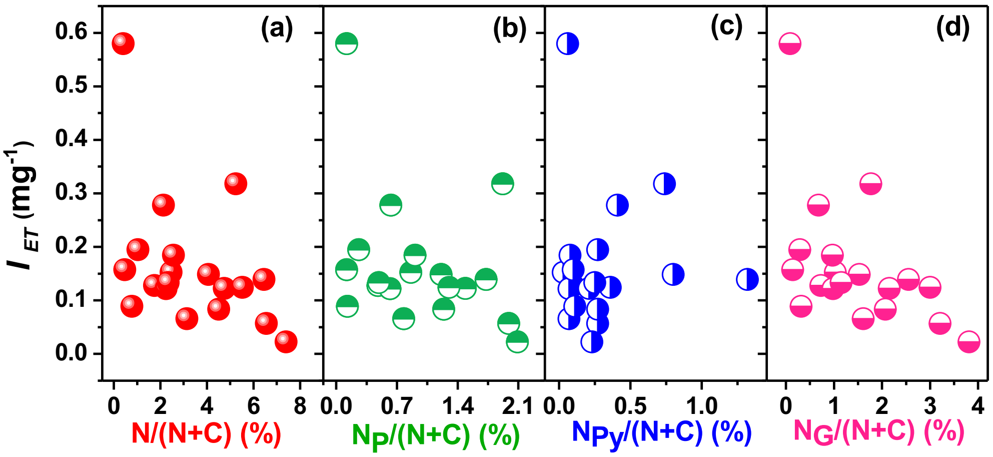

Figure S4. Dependences of the intensity of electron transfer on the content of (a) N/(N+C), (b) N_P/(N+C), (c) N_{Py}/(N+C), and (d) N_G/(N+C) of N-doped carbons.

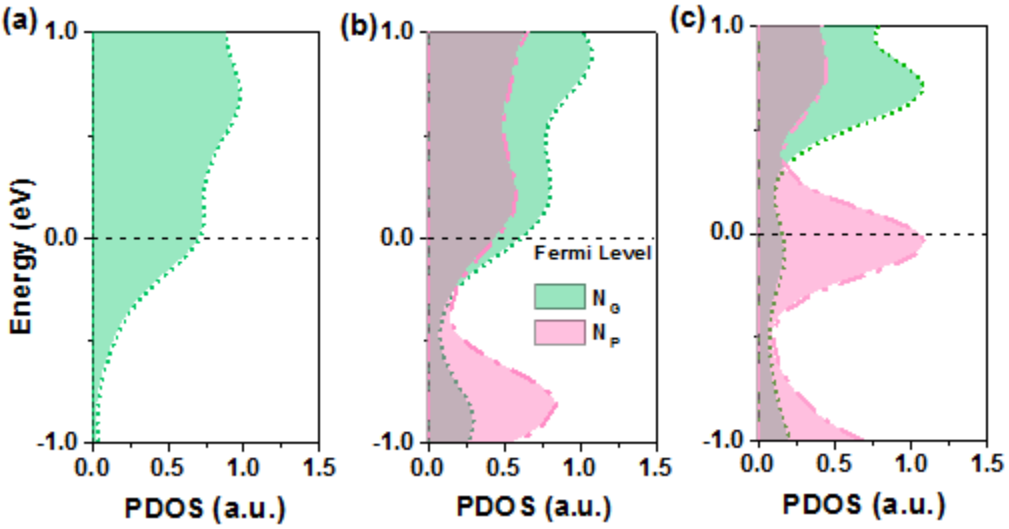

Figure S5. The partial density of states (PDOS) of N_G and N_P atoms for N-doped graphene (a) containing pure N_G, (b) with [N_P]:[N_G]=1:2, (c) with [N_P]:[N_G] =1:1, respectively.

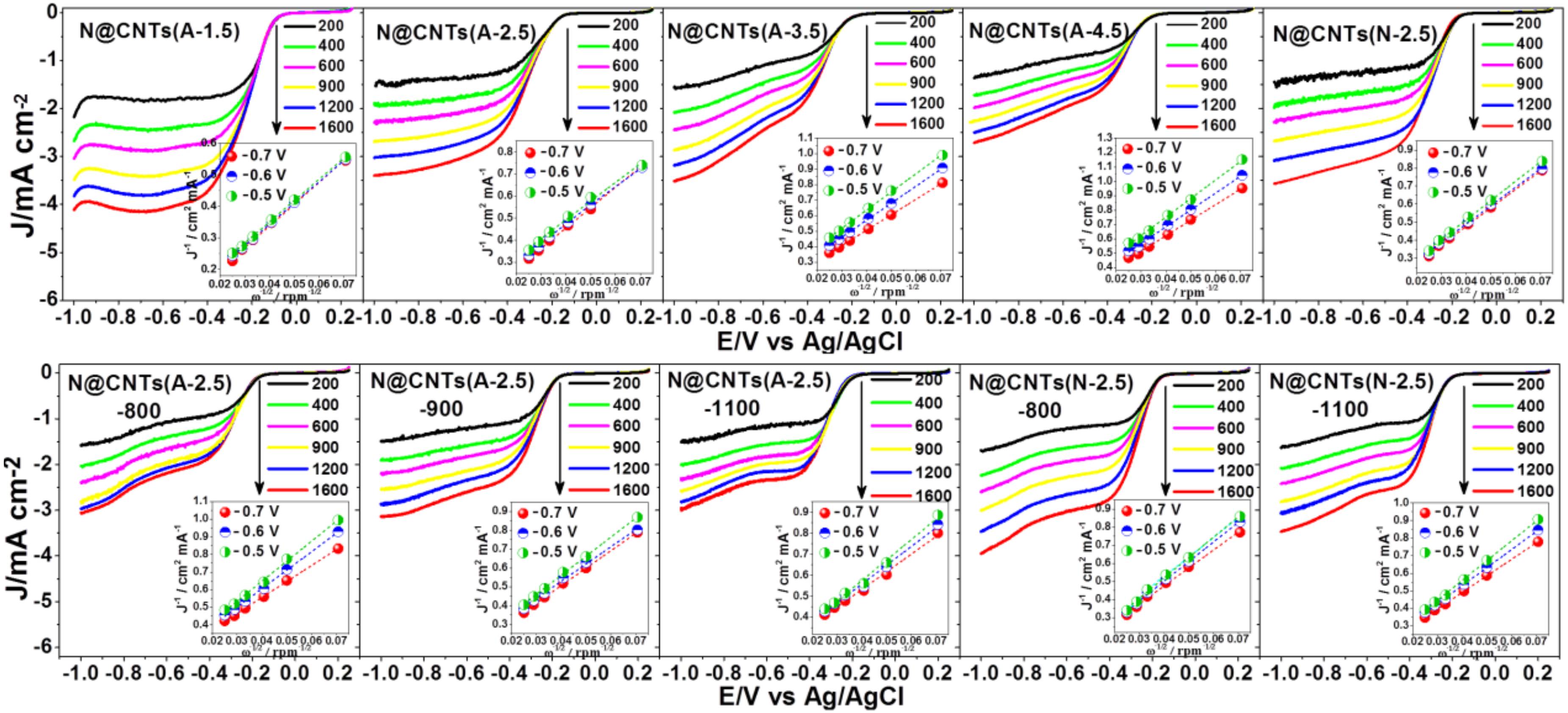

Figure S6. LSV polarization curves for ORR on N@CNTs with different rotation speeds. The insets show the corresponding K-L plots at -0.7 V, -0.6 V, -0.5 V.

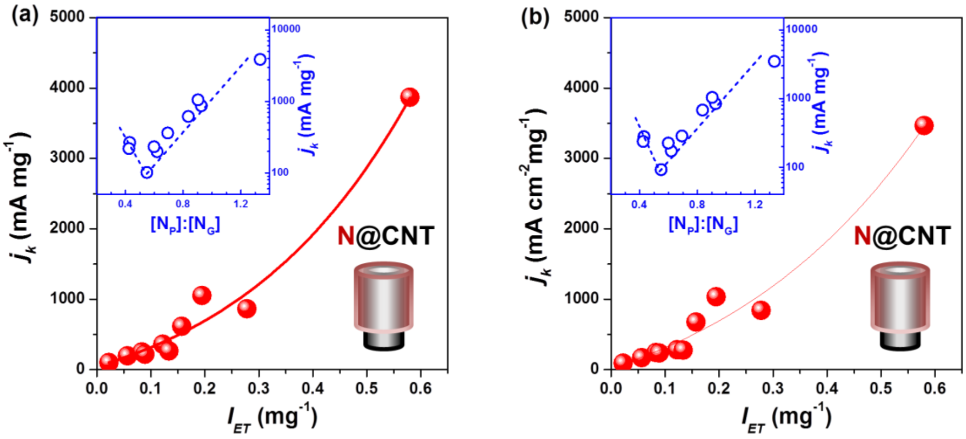

Figure S7. Dependences of kinetic current densities (j_k) of ORR at (a) -0.6 V, and (b) -0.5 V vs Ag/AgCl on the intensity of electron transfer and [N_P]:[N_G] ratio (inset) of N@CNTs samples with a coaxial cable structure as shown at lower right panel. j_k was obtained according to the Koutecky-Levich (K-L) equation, j^{-1} = $(0.62nFCD^{2/3}\upsilon^{-1/6}\omega^{1/2})^{-1}$ + j_k^{-1}, and normalized by the mass of NCs. Reaction conditions: the linear scan voltammetric tests for ORR were performed in O_2-saturated 0.1 M KOH from -1 V to 0.2 V at the scan rate of 5 mV s^{-1} under different rotation rates.

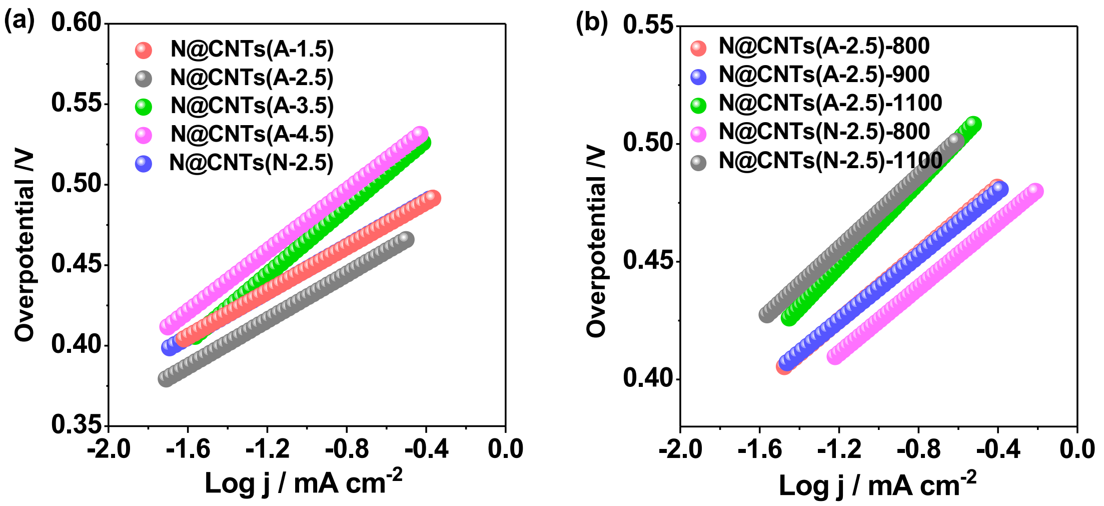

Figure S8. The Tafel curves of N@CNTs samples.

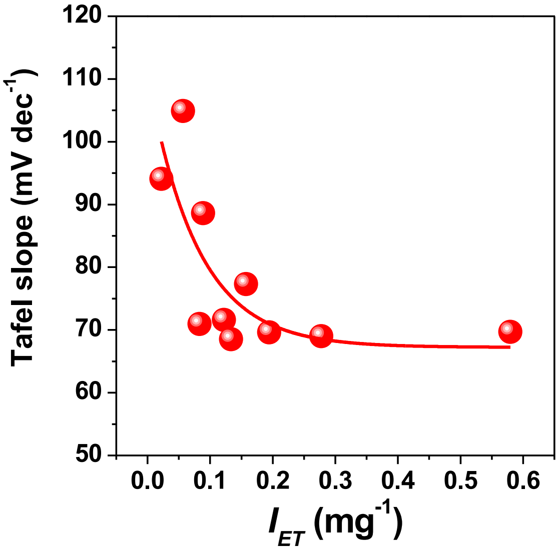

Figure S9. Dependence of Tafel slope on the intensity of electron transfer.

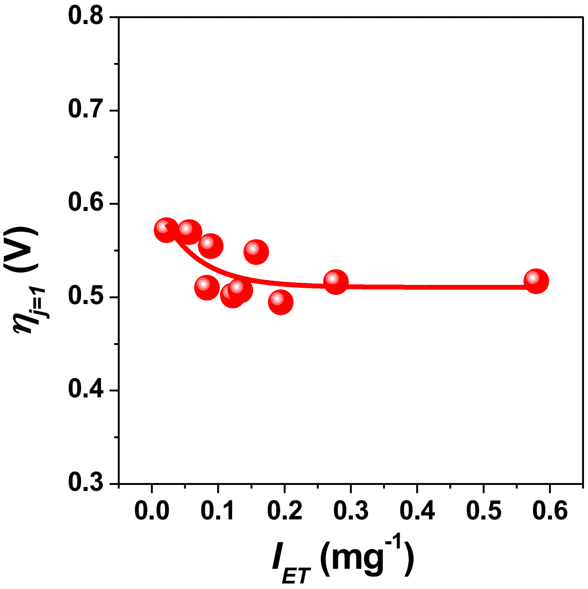

Figure S10. Dependence of overpotential at j=1 on the intensity of electron transfer.

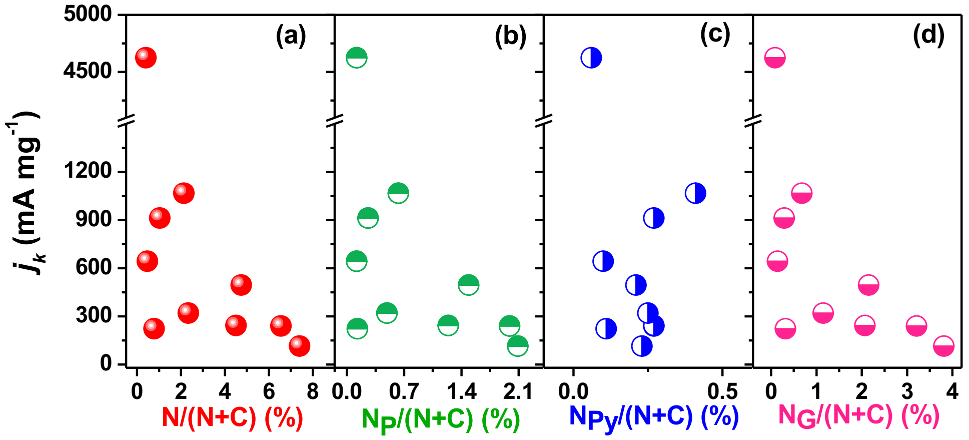

Figure S11. Dependence of kinetic current densities (j_k) of ORR at - 0.7 V on the content of (a) N/(N+C), (b) N_P/(N+C), (c) N_{Py}/(N+C), and (d) N_G/(N+C) of N-doped carbons.
